# More knee reinjuries after ACL reconstruction compared to nonsurgical treatment of the ACL. A 2‐year follow‐up of the NACOX prospective cohort study

**DOI:** 10.1002/ksa.12473

**Published:** 2024-10-03

**Authors:** Anna S. Selin, Håkan Gauffin, Henrik Hedevik, Anne Fältström, Joanna Kvist

**Affiliations:** ^1^ Department of Orthopaedics and Department of Biomedical and Clinical Sciences Linköping University Linköping Sweden; ^2^ Department of Health Center for Medical Image Science and Visualization (CMIV), Medicine and Caring Sciences, Linköping University Sweden; ^3^ Unit of Physiotherapy, Department of Health Medicine and Caring Science, Linköping University Linköping Sweden; ^4^ Region Jönköping County, Rehabilitation Centre, Ryhov County Hospital Jönköping Sweden

**Keywords:** meniscus, nonsurgical treatment, rehabilitation, return to sport

## Abstract

**Purpose:**

To describe knee reinjuries and surgeries within 2 years after an acute anterior cruciate ligament (ACL) injury, in patients treated with or without ACL reconstruction (ACLR).

**Methods:**

Two years follow‐up of 275 patients (52% females, mean age 25.2 [SD 7.0] years) about knee reinjuries and surgeries were analysed from the prospective NACOX cohort study, aiming to describe recovery after an acute ACL injury treated according to clinical routine. Knee reinjury was defined as increase or new symptoms due to new trauma. At 2 years after injury, 169 (61%) had undergone an ACLR. Results are presented with descriptive statistics and risk ratios (RR).

**Results:**

Thirty‐two patients sustained reinjuries within 2 years; 6 in the non‐ACLR group, and 26 in the ACLR group (7 before and 19 after ACLR). Twelve patients in the ACLR group sustained a graft rupture and three did an ACL revision. Patients with non‐ACLR, aged 21–25 years, had a 5.9‐fold higher risk for reinjury than those aged 15–20 years (RR 5.9 [1.3–26.9]; *p* = 0.012). Twenty‐four patients had surgery in the non‐ACLR group and 36 patients in the ACLR group (excluding primary ACLR), where six were before ACLR and 30 were after ACLR.

**Conclusion:**

Twelve percent sustained a knee reinjury and 21% underwent knee surgery within 2 years after the index ACL injury. There was a higher reinjury incidence in the ACLR group, but no difference in incidence of surgeries. Thus, ACLR did not reduce the risk of traumatic reinjuries or surgeries, which is important for treatment decision considerations.

**Level of Evidence:**

Level I.

**Abbreviations**
ACL: anterior cruciate ligament
ACLR: anterior cruciate ligament reconstruction
BMI: body mass index
CI: confidence interval
IKDC: International Knee Documentation Committee
MRI: magnetic resonance imaging
RR: risk ratio
RTS: return to sports
WHO: World Health Organization

## INTRODUCTION

Anterior cruciate ligament (ACL) injury is a common and severe knee injury occurring mainly in young physically active athletes in pivoting sports. The treatment after an ACL injury can be rehabilitation alone or in combination with ACL reconstruction (ACLR) and aims to restore knee function, prevent new injuries, and in many cases makes it possible for the athlete to return to sports [3].

The clinical, functional, and return‐to‐sports outcomes may not differ between surgically or nonsurgically treatment of the ACL injury [6, 11, 24]. Yet, one argument for having an ACLR is the risk of knee reinjuries (i.e., new knee injuries to the same knee), especially meniscal injuries, if no ACLR is performed. Previous studies report a high risk (10%–72%) of meniscal injuries or meniscal surgeries after the initial ACL injury [3, 8, 11, 18, 20, 21, 22] and that risk is associated with persistent knee instability [3, 28]. Early ACLR is suggested to decrease that risk for reinjuries [3, 27, 28]. However, a problem with previous studies is the selection bias, where patients are mostly included because they have a late ACLR due to knee problems or reinjuries and patients who function well after nonsurgically treated ACL are not followed up [4]. Evidence from the two randomized controlled trials (RCT) [6, 31], comparing early ACLR with rehabilitation alone plus optional ACLR, show no difference in the number of meniscus surgeries between groups at 2‐ [31] and 5‐ [6] year follow‐ups. An additional issue with previous studies is that they do not differentiate for meniscal surgeries performed due to concomitant meniscus injury at the index ACL injury, that is, not related to a new trauma or count for meniscal surgeries performed at the same time as an early ACLR. Approximately half of the patients have concomitant meniscal injuries at index ACL injury [1, 19] and one‐third of the patients have a meniscal procedure during the ACLR [31]. Thus, meniscal surgeries may have been overestimated in patients treated without ACLR and underestimated in patients treated with ACLR.

On the other hand, an early ACLR, within 3 months from ACL injury, does not prevent reinjuries or surgeries. The risk for revision surgery is 2.3% within 2 years and 5.8% in 5 years in a general population [5], and the risk for graft rupture after ACLR is between 10% and 27% [7, 8, 18, 32]. Risk factors for ACL graft ruptures or contralateral ACL injury include young age, return to high level of activity, time to return to sports, and graft type in ACLR [2, 13, 15, 23, 32]. Previous studies also present high risk for knee reinjuries after ACLR (e.g., meniscus injuries), ranging between 34% and 56% [8, 11] and the risk for other knee surgeries (e.g., screw removal, meniscus procedures, cyclops removal) being 16% [20].

Thus, the reinjury panorama after an ACL injury is not well described, especially following a nonsurgical treatment where systematic follow‐ups are sparse. This is important for clinicians in the decision for treatment of an ACL injury, whether to do an ACLR or not. The aim of this study was to describe the rate and nature of traumatic knee reinjuries and surgeries (excluding primary ACLR) of the index knee within 2 years after an acute ACL injury in both patients with surgical, that is, ACLR, and nonsurgical treatment of the ACL. In addition, the study aims to investigate factors that may predict the risk for knee reinjuries or surgeries. Our hypothesis was that patients treated with rehabilitation alone will have higher incidence in both reinjuries and surgeries.

## MATERIALS AND METHODS

### Study design and patients

This study is based on the cohort and data from the NACOX study (Natural Corollaries and Recovery after an acute ACL injury) [17], which is a multicenter, prospective cohort study including patients from six sites in Sweden (mix of public and private healthcare clinics), with an acute ACL injury between May 2016 and October 2018. The inclusion criteria were patients between 15 and 40 years old with an acute ACL injury (within 6 weeks). The exclusion criteria were a previous ACL injury on the same knee, serious concomitant injury (fracture that requires separate treatment), inability to understand written and spoken Swedish, cognitive impairment and other illness or injury that impairs function (e.g., fibromyalgia, rheumatic diseases and other diagnoses associated with chronic pain). Participation in the study did not alter the usual course of treatment for patients with an ACL injury at recruiting centres [17]. Consequently, the patients in this cohort had individualized knee rehabilitation and had either ACLR at some point after the index ACL injury or remained treated without ACLR, according to a shared decision‐making that we have described previously for a subgroup of the patients [9, 10]. Patients' appraisal of rehabilitation and adherence was higher post‐ACLR compared to the patients who had a non‐ACLR treatment [29]. The main reasons for having ACLR were high activity demands (i.e., want to return to pivoting sports) and knee instability, whereas the main reason for having a non‐ACLR treatment was no knee instability and no problems with knee function [10]. All patients received study information and gave their consent to participate in this study. The Swedish Ethical Review Authority has approved the NACOX study (Dnr. 2016/44‐31 and 2017/221‐32).

### Data collection

Patients included in the NACOX cohort had a clinical evaluation by an orthopaedic surgeon and underwent a magnetic resonance imaging (MRI) at baseline to confirm the ACL rupture and identify concomitant injuries to the knee [1]. Patients were closely monitored with continuous registration, follow‐ups, and evaluation of knee function, symptoms, activity participation, and reinjuries or surgeries, as previously described [17]. Digital questionnaires were sent out regularly (initially weekly, later monthly) and if new injury occurred, patients were followed up by telephone contact. Medical charts and radiology were reviewed to get more information about any reinjury or knee surgery. Reports for activity level and sport participation at each follow‐up were classified according to the Tegner score [30] and International Knee Documentation Committee (IKDC) level (level I pivoting and contact sports, level II pivoting noncontact sports, and level III neither pivoting nor contact sports) [12, 14]. The timing for return to sports was when (months from initial ACL injury) the patient reported that he/she participated at the same IKDC level as before the injury.

If patients failed to respond to the questionnaires at 2 years follow‐up, we contacted them by telephone (call and/or SMS), letter, or mail. Medical charts were reviewed to see if the patient had visited a healthcare clinic due to knee problems. In addition, patients were checked on the Swedish National ACL register, where more than 90% of the ACLR in Sweden are registered [5], to see if they had undergone any additional surgeries. For 11 patients (4%), we got no answer from surveys or telephone, and there was no information about reinjuries or surgeries in the medical charts nor in the registry, so we interpreted that these patients had no reinjury or knee surgery.

### Classification of knee reinjuries and surgeries

We defined knee reinjury as in increase of knee symptoms or new symptoms from the knee as a result of a traumatic injury during an activity‐related exposure [26]. All reported reinjuries were classified as reinjury not leading to surgery or reinjury leading to surgery. In addition, graft ruptures were reported separately (graft ruptures were excluded from previous categories).

Knee surgeries were grouped as:

- ACLR
- Diagnostic arthroscopy
- Arthroscopic meniscectomy
- Arthroscopic meniscal repair
- Arthroscopic debridement, for example, due to cartilage lesions (not infection)
- Extraction of osteosynthesis material from ACLR (open or arthroscopic surgery)
- Arthroscopic lavage and debridement due to infection post‐ACLR
- Microfracturing
- ACL Revision

Only surgeries performed within 2 years from the index ACL injury are reported. If one reinjury was classified as ‘reinjury leading to surgery’ but the surgery was performed more than 2 years after the index ACL injury, the surgery is not reported under surgeries.

### Statistical analysis

All statistical analyses were performed with IBM SPSS Statistics for Windows, v29. Descriptive statistics of patient's characteristics are presented as mean ± standard deviation or median with interquartile range or number and percentages.

Patients were grouped relating to ACLR. Non‐ACLR are patients who did not undergo an ACLR within 2 years from index ACL injury. ACLR were patients who did undergo ACLR within 2 years from index injury. Reinjuries and surgeries are reported and described for the whole cohort and separately in the two groups, non‐ACLR and ACLR (including reinjuries and surgeries both before and after ACLR).

Patient characteristics were compared between the groups non‐ACLR and ACLR with the *t*‐test for variables on continuous scales [age and body mass index (BMI)] or the Mann–Whitney *U*‐test for variables on ordinal scale (Tegner activity level scale). Nominal variables were compared with the chi‐square test (age intervals, sex, and concomitant injuries) or the Fishers' exact test (BMI intervals and sports within IKDC level I before injury) when more than 20% of expected cells were less than 5.

Risk of reinjuries or surgeries was calculated for the independent variables; sex (male/female), age (15–20, 21–25, and 26–40 years), BMI (<25, 25–29.9, and ≥30 kg/m^2^), concomitant injuries (yes/no), and preinjury sports level (IKDC‐level I, II–III). Differences within each independent variable were analysed with Generalized Linear Model on the binomial distributed outcome variables (reinjury or surgery) using log link function. Results of the models are presented as risk ratio (RR) with a 95% confidence interval (CI) for the cohorts: (1) non‐ACLR or before ACLR and (2) after ACLR.

The significance level is at *p* < 0.05.

## RESULTS

### Patient characteristics

**Figure 1:**
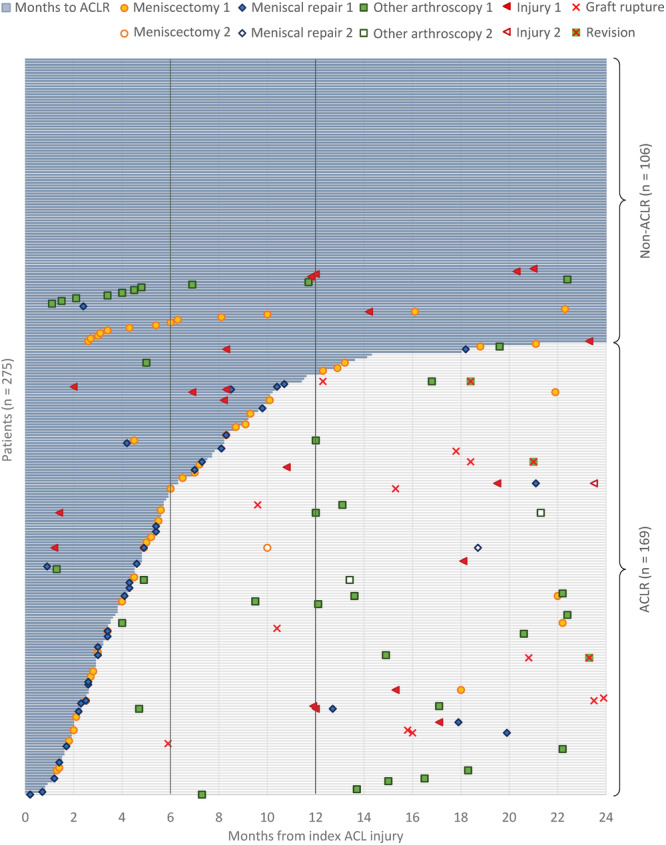
Overview of reinjuries and surgeries within 2 years from index ACL injury. All 275 included patients are represented with a line on the *y*‐axis, each patient has its own line. The blue line changes to gray line at the time (*y*‐axis) when an ACLR is performed (patients on the top of the diagram having only blue lines are treated with no ACLR). The *x*‐axis is the follow‐up time from 0 to 24 months from baseline. The figure shows each patient's events (surgeries, injuries) and the time for the events.

Descriptive data for the baseline characteristics are reported in Table 1. There were 275 patients included in the study. Ninety percent of patients recruited from three sites and the rest 10% from the remaining three sites. The majority of patients were active in contact or pivoting sports before to injury and almost all (*n* = 259, 94%) had the goal to return to sport. At 2 years after injury, 169 (61%) had undergone an ACLR in a mean of 6.8 (SD 6.7) months after the injury (Figure 1). Hamstrings graft was used in 149 (88%) patients, patellar tendon in 18 (11%) patients, and quadriceps tendon in 2 (1%) patients.

**Table 1:** Patient characteristics.

	Total *N* = 275	Non‐ACLR *N* = 106	ACLR *N* = 169	*p* Values
Age, years	25.2 ± 7.0	27.6 ± 7.1	23.8 ± 6.6	<0.001
Age interval				0.002
15–20 years	96 (35)	24 (23)	72 (43)	
21–25 years	65 (24)	24 (23)	41 (24)	
26–30 years	50 (18)	24 (23)	26 (15)	
31–40 years	64 (23)	34 (32)	30 (18)	
Sex				0.129
Male	132 (48)	57 (54)	75 (44)	
Female	143 (52)	49 (46)	94 (56)	
BMI	23.9 ± 3.2	24.4 ± 3.7	23.6 ± 2.8	0.319
BMI classification (WHO)				0.167
<18.5 Underweight	2 (1)	2 (2)	0	
18.5–24.9 Healthy weight	193 (70)	69 (65)	124 (74)	
25.0–29.9 Overweight	67 (25)	29 (28)	38 (23)	
≥30.0 Obesity	12 (4)	6 (6)	6 (4)	
Concomitant injuries^a^	189 (74)	77 (75)	112 (74)	0.916
Meniscus injury	164 (65)	63 (62)	101 (67)	0.403
Other ligament rupture	44 (17)	25 (24)	19 (13)	0.016
Cartilage injury	34 (13)	19 (18)	15 (10)	0.050
Preinjury Tegner activity level, median (IQR)	7.0 (4.0, 9.0)	6.5 (4.0, 9.0)	8.0 (4.0, 9.0)	<0.001
Preinjury IKDC activity‐level sports^b^				<0.001
Level I	158 (57)	45 (42)	113 (67)	0.293
Football	99 (63)	28 (62)	71 (63)	
Floorball	32 (20)	12 (27)	20 (18)	
Handball	17 (11)	2 (4)	15 (13)	
Other contact/pivoting sports^c^	10 (6)	3 (7)	7 (6)	
Level II	34 (12)	16 (15)	18 (11)	
Level III	83 (30)	45 (42)	38 (22)	

*Note*: Data are presented as *n* (%) or mean ± SD unless otherwise indicated.

Abbreviations: ACLR, anterior cruciate ligament reconstruction; BMI, body mass index; IKDC, International Knee Documentation Committee; WHO, World Health Organization.

^a^ Concomitant injuries (medial and/or lateral meniscus, partial or total ligament (MCL and/or LCL) rupture, partial or full cartilage lesion) in the ipsilateral knee joint. Available data from 254 patients.

^b^ IKDC‐level, I: contact and pivoting sports, II: noncontact but pivoting sports, III: neither contact nor pivoting sports.

^c^ Basket, Ice hockey, American football, Rugby.

### Knee reinjuries

**Figure 2:**
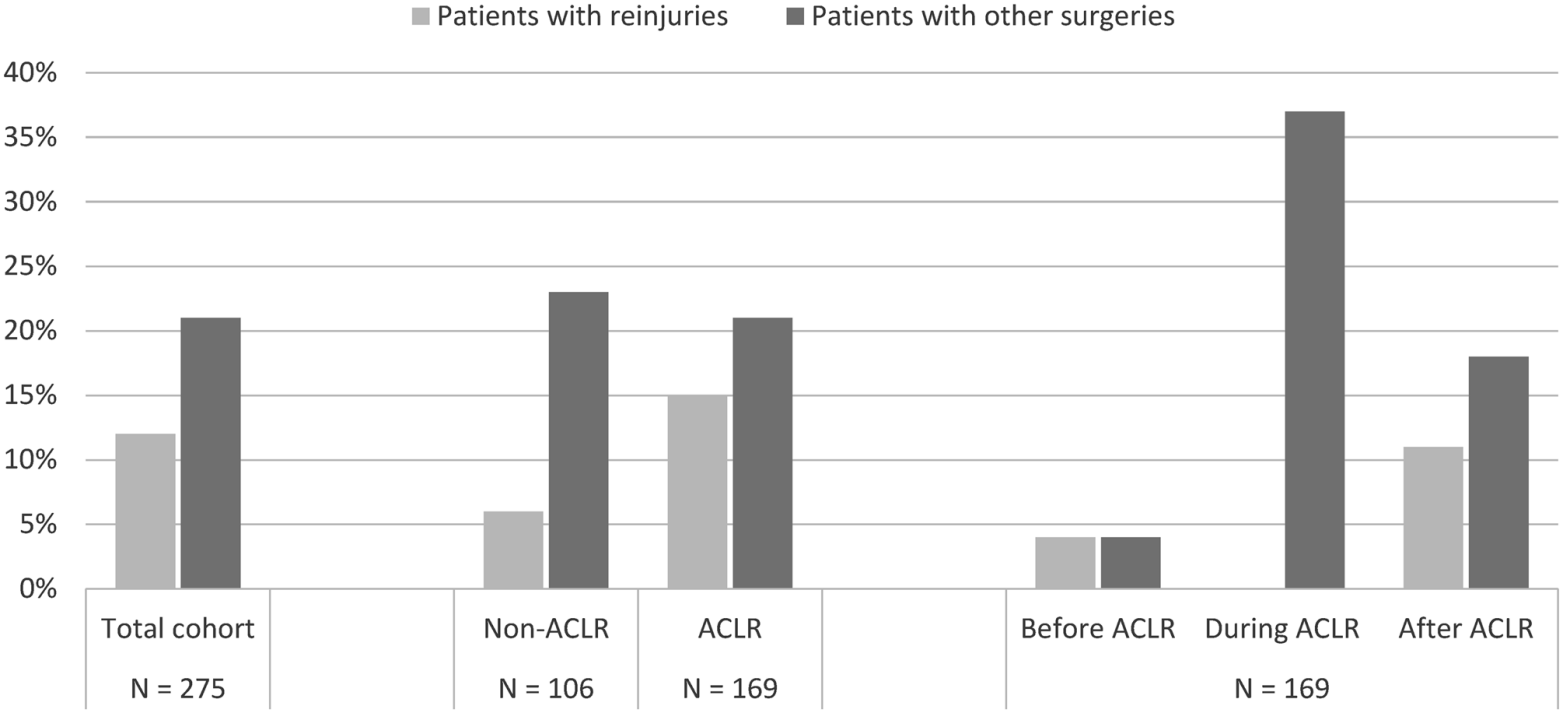
Percentage of reinjuries and surgeries [other than anterior cruciate ligament reconstruction (ACLR)] in the: total cohort (*n* = 275), non‐ACLR (*n* = 106) and ACLR (*n* = 169) cohorts and ACLR cohort (*n* = 169) divided for reinjuries and surgeries that occurred before, during, and after the index ACLR.

Reinjuries are reported in Table 2 and presented in Figures 1 and 2. There were 32 (12%) patients who sustained reinjuries within 2 years of index injury. Six of them were in the non‐ACLR group and 26 in the ACLR group, of whom seven patients got the injury before ACLR, and 19 got the injury after ACLR. Of the seven patients with a reinjury before the ACLR, two got reinjured when playing football (7 resp. 8 months post index ACL injury) and the remaining five during activities of daily living (e.g., fell when playing with kids, slipped when walking, climbing a bicycle). Twelve of the 19 injuries that occurred after an ACLR were graft ruptures. One patient had two reinjuries within 2 years of the index injury, both occurred after ACLR and were classified as reinjury leading to surgery.

**Table 2:** Description of reinjuries and if the injury occurred before or after return to sports.

	Total *N* = 275	Non‐ACLR *N* = 106	ACLR *N* = 169	
Patients with no reinjury	243_/275_ (88)	100_/106_ (94)	143_/169_ (85)	
			Reinjury before ACLR	Reinjury after ACLR
Patients with reinjuries	32_/275_ (12)	6_/106_ (6)	7_/169_ (4)	19_/169_ (11)
Months to reinjury, m ± SD	13.5 ± 6.3	17.1 ± 5.0	5.2 ± 3.5	15.5 ± 4.8
Injury not leading to surgery^a^	4_/32_ (13)	2^b^_/6_ (33)	0_/7_	2_/19_ (11)
Injury leading to surgery	16_/32_ (50)	4_/6_ (67)	7_/7_ (100)	5_/19_ (26)
ACLR	8_/16_ (50)	2^b^_/4_ (50)	6_/7_ (86)	n/a
Meniscectomy	3_/16_ (19)	2_/4_ (50)	0_/7_	1_/5_ (20)
Meniscal repair	4_/16_ (25)	0_/4_	1_/7_ (14)	3_/5_ (60)
Debridement	1_/16_ (6)	0_/4_	0_/7_	1_/5_(20)
Graft rupture	12_/32_ (38)	n/a	n/a	12_/19_ (63)
Revision	3_/12_ (25)	n/a	n/a	3_/12_ (25)
No RTS before reinjury	12_/32_ (38)	2_/6_ (33)	5_/7_ (71)	5_/19_ (26)
RTS before reinjury	20_/32_ (63)	4_/6_ (67)	2_/7_ (29)	14_/19_ (74)
Level I	17_/20_ (85)	4_/4_ (100)	1_/2_ (50)	12_/14_ (86)
Level II	3_/20_ (15)	0_/4_	1_/2_ (50)	2_/14_ (14)
Level III	0_/20_	0_/4_	0_/3_	0_/14_

*Note*: Data are presented as n (%).

Abbreviations: ACLR, anterior cruciate ligament reconstruction; m ± SD, mean ± standard deviation; n/a, nonapplicable; RTS, return to sport.

^a^ The four injuries not leading to surgery were giving ways in the non‐ACLR group and one patella subluxation and one rotational trauma with the foot fixed on the ground in the ACLR group.

^b^ Two patients had a reinjury leading to ACLR, but the ACLR was after 2 years.

Twenty of the 32 patients (63%) in the whole cohort with reinjuries had return to sports (RTS) before the reinjury, 85% of them to level I sport. In the non‐ACLR group, four of six patients (67%) had RTS before the reinjury, all to IKDC level I sports (all to football). In the ACLR group, 16 of 26 (65%) of the patients had RTS before the reinjury, 13 of them (81%) to IKDC level I (four patients to football, three to handball, three to floorball, one to basketball, one to martial arts, and one to alpine skiing) (Table 2).

Patients with non‐ACLR or before the ACLR aged 21‒25 years had a 5.9‐fold higher risk of knee reinjuries than younger patients [RR 5.9 (1.3‒26.9); *p* = 0.012]. There was no statistically significant difference between other different stratified baseline factors (sex, BMI, preinjury activity level) (Table 3).

**Table 3:** Risk analyses for reinjuries and surgeries for the independent variables sex, age, BMI, concomitant injuries and preinjury activity level for the cohorts (1) non‐ACLR or before the ACLR and (2) after ACLR.

	Reinjuries in non‐ACLR or before ACLR		Reinjuries after ACLR		Other surgeries in non‐ACLR or before ACLR		Other surgeries after ACLR	
	*N* = 275		*N* = 169		*N* = 275		*N* = 169	
	*n* (%)	RR (95% CI)	*n* (%)	RR (95% CI)	*n* (%)	RR (95% CI)	*n* (%)	RR (95% CI)
Sex		*p* = 0.891		*p* = 0.444		*p* = 0.317		*p* = 0.090
Male (ref)	6 (5)		10 (13)		17 (13)		9 (12)	
Female	7 (5)	1.1 (0.3–3.1)	9 (10)	0.7 (0.3–1.7)	13 (10)	0.7 (0.4–1.4)	21 (22)	1.9 (0.9–3.8)
Age interval		***p*** = **0.012**		*p* = 0.680		*p* = 0.418		*p* = 0.455
15–20 years (ref)	2 (2)		8 (11)		7 (7)		13 (18)	
21–25 years	8 (12)	5.9 (1.3–26.9)	6 (15)	1.3 (0.5–3.5)	8 (12)	1.7 (0.6–4.4)	5 (12)	0.7 (0.3–1.8)
26–40 years	3 (3)	1.3 (0.2–7.4)	5 (9)	0.8 (0.3–2.3)	15 (13)	1.8 (0.8–4.2)	12 (2%)	1.2 (0.6–2.4)
BMI classification		*p* = 0.835		*p* = 0.822		*p* = 0.925		*p* = 0.279
Under/healthy weight^a^ (ref)	9 (5)		13 (10)		21 (11)		19 (15)	
Overweight	3 (4)	1.0 (0.3–3.4)	5 (13)	1.3 (0.5–3.3)	8 (12)	1.1 (0.5–2.4)	9 (24)	1.5 (0.8–3.1)
Obese	1 (8)	1.8 (0.2–13.1)	1 (17)	1.6 (0.2–10.2)	1 (8)	0.8 (0.1–5.3)	2 (33)	2.2 (0.7–7.3)
Concomitant injury		*p* = 0.660		*p* = 0.267		*p* = 0.065		*p* = 0.137
No (ref)	4 (6)		2 (5)		3 (5%)		10 (26)	
Yes	9 (5)	0.8 (0.2–2.4)	13 (12)	2.3 (0.5–10.0)	26 (14)	3.0 (0.9–10.0)	17 (15)	0.6 (0.3–1.2)
Preinjury IKDC‐level		*p* = 0.870		*p* = 0.710		*p* = 0.564		*p* = 0.470
Level I (ref)	10 (6)		14 (12)		20 (13)		19 (17)	
Level II	3 (9)	1.4 (0.4–4.8)	1 (6)	0.4 (0.1–3.2)	3 (9)	0.7 (0.2–2.2)	2 (11)	0.7 (0.2–2.6)
Level III	0	NA	4 (5)	0.8 (0.3–2.4)	7 (8)	0.7 (0.3–1.5)	9 (24)	1.4 (0.7–2.8)

*Note*: Bold *p* values indicate statistically significant between group differences. Abbreviations: ACLR, anterior cruciate ligament reconstruction; BMI, body mass index; CI, confidence interval; IKDC, International Knee Documentation Committee; RR, risk ratio.

^a^ Underweight and healthy weight were merged because of low number in underweight.

### Surgeries

Surgeries are reported in Table 4 and presented in Figures 1 and 2. A total of 59 (21%) patients had a knee surgery (excluding primary ACLR) within 2 years from the index ACL injury.

**Table 4:** Description of knee surgeries, excluding primary ACLR.

	Total *N* = 275	Non‐ACLR *N* = 106	ACLR *N* = 169		
Patients with no other surgery	216_/275_ (79)	82_/106_ (77)	134_/169_ (79)		
			Other surgeries before ACLR	Additional surgical treatment during ACLR	Other surgery after ACLR
Patients with other surgeries	59_/275_ (21)	24_/106_ (23)	6^a^_/169_(4)	63_/169_ (37)	30^a^_/169_ (18)
Number of other surgeries	67	24	6	63	37
Type of surgery					
Diagnostic	8_/67_ (12)	6_/24_ (25)	0_/6_	n/a	2_/37_ (5)
Meniscectomy	19_/67_ (28)	13_/24_ (54)	1_/6_ (17)	31_/63_ (49)	5_/37_ (14)
Meniscal repair	9_/67_ (13)	1_/24_ (4)	3_/6_ (50)	29_/63_ (46)	5_/37_ (14)
Debridement (Shaving)	16_/67_ (24)	4_/24_ (17)	2_/6_ (33)	3_/63_ (5)	10_/37_ (27)
Extraction of osteosynthesis	8_/67_ (12)	n/a	n/a	n/a	8_/37_ (22)
Lavage due to infection	3_/67_ (4)	0_/24_	0_/6_	n/a	3_/37_ (8)
Microfracturing	1_/67_ (1)	0_/24_	0_/6_	0_/63_	1_/37_ (3)
Revision	3_/67_ (4)	n/a	n/a	n/a	3_/37_ (8)

*Note*: Data are presented as *n* (%).

Abbreviation: ACLR, Anterior cruciate ligament reconstruction.

^a^ One patient had surgery both before and after ACLR.

In the non‐ACLR group, 24 (23%) patients had a surgery, the majority being meniscectomies (*n* = 13, 54%), where only two were due to traumatic reinjury (Table 2). Nine (64%) of the total 14 meniscectomies/meniscal repairs had meniscus injuries that were visible on MRI after the index ACL injury, that is, concomitant injuries to the index ACL injury (Table 5). The time between index ACL injury and meniscus surgeries was in the non‐ACLR group 7.2 months (SD 5.9, *n* = 13) for meniscectomies and 2.4 months (*n* = 1) for meniscus repair. In the ACLR group, before the ACLR surgery, one meniscectomy was performed 4.5 months after index ACL injury and the three meniscal repairs were performed 4.5 (SD 3.8) months after index ACLR. In the ACLR group, after the ACLR surgery, five meniscectomies were performed 18.2 (SD 5.2) months after index ACL injury and the five meniscal repairs were performed 18.1 (SD 3.2) months after index ACLR (Figure 1).

**Table 5:** Patients with meniscus surgery for a meniscal injury that was visible on baseline MRI, acute after the index ACL injury.

Meniscus surgery, visual meniscus injury at baseline	Total *N* = 26	Non‐ACLR *N* = 14	ACLR *N* = 12	
			New surgery before ACLR	New surgery after ACLR
Yes	18/_26_ (69)	9/_14_ (64)	1/_3_ (33)	8/_9_ (89)
No	8/_26_ (31)	5/_14_ (36)	2/_3_ (67)	1/_9_(11)

*Note*: Data are presented as *n* (%).

Abbreviations: ACL, anterior cruciate ligament; ACLR, anterior cruciate ligament reconstruction; MRI, magnetic resonance imaging.

In the ACLR group, 36 (21%) patients had a surgery, six (4%) before the ACLR and 30 (18%) after ACLR (one patient had surgery both before and after ACLR). The most common surgery after ACLR was debridement (27%). There were three (8%) ACLR revisions. During the ACLR, additional surgical procedures were performed in 63 patients (37%), and the most common procedures were meniscectomies (49%) and meniscal repair (46%) (Table 4).

There were eight patients who had two surgeries within 2 years, all were in the ACLR group, after the ACLR. Therefore, the total number of surgeries in both groups within 2 years was 67; 24 in the non‐ACLR group and 43 in the ACLR group. Of the total 67 knee surgeries, 28 (41%) were meniscal surgery in 27 patients (one patient had both meniscal repair and meniscectomy). Eighteen of these had the meniscal injury visible on MRI at the baseline (missing data in one patient due to no available MRI from baseline). More information can be found in Table 5.

There were no statistically significant risk ratios for new surgeries for the predefined independent factors sex, age, BMI, concomitant injuries, or preinjury sports level (Table 3).

## DISCUSSION

This study shows that 12% of all patients sustained a traumatic knee reinjury during an activity‐related exposure that resulted in increase of knee symptoms or new symptoms from the knee within 2 years from index ACL injury. The highest occurrence of knee reinjury was in the ACLR group after ACLR (11%) and lowest in the non‐ACLR group (6%). One in five patients underwent a knee surgery within 2 years from index ACL injury (excluding the primary ACLR), with similar results in the non‐ACLR group and ACLR group. In addition, more than a third of the patients had additional surgical treatment to other structures in the knee, most common meniscus surgery, during the ACLR. Patients aged between 21 and 25 years with no ACLR or before an ACLR had a higher risk for knee reinjuries compared to patients aged 15‒20. Our hypothesis, that ACLR will reduce the risk of reinjuries, and surgeries was not proven.

The higher incidence of knee reinjuries in the ACLR group is in contrast with previous studies reporting a higher risk for knee reinjuries in patients without ACLR [16, 18, 27, 28]. Our study is unique to our knowledge, following a population for 2 years after acute ACL injury, without influencing treatment choice (i.e., ACLR or not). Previous studies are either RCT [27] or systematic reviews including studies with higher risk of bias [16, 18, 28]. In our study, higher number of patients in the ACLR group were active in pivoting sport and had a higher Tegner score before the ACL injury, compared to the non‐ACLR group. This may influence the risk of reinjuries if patients return to their previous sports activity. Thus, it is important to consider a possible selection bias as discussed both by Lien‐Iversen [18], Melbye et al. [22], and Kopershoek et al. [16], where patients who return to pivoting sports are at a higher risk for reinjuries [7, 32]. However, in our study, the percentage of patients who had a reinjury after RTS were similar between treatment groups, and therefore RTS could not explain the higher prevalence of reinjuries in the ACLR group. In addition, our risk analysis showed that preinjury activity level did not influence the risk for reinjuries.

The only factor that could predict knee reinjuries in our study was age, where being 21‒25 years of age increased the risk for knee reinjury by almost six times compared to a younger age in the group non‐ACLR and before ACLR. It is interesting that no factors predicted knee reinjuries and surgeries in the ACLR group. Previous studies lack risk analyses of factors for reinjuries in both patients treated with or without ACLR. One study reports a two‐ to five‐times higher risk for knee reinjuries and surgeries in female football players aged 16–25 with ACLR who returned to football compared to players who did not return and the control group [7]. Other factors that may influence the risk for reinjury could be the rehabilitation protocols, including the return‐to‐sports criteria. In our study, patients received standard care according to clinical routine and guidelines, meaning that the rehabilitation was individualized for each patient. Our patients were treated by 78 different physiotherapists, where 94% of them had more than 6 years' experience treating patients with ACL injuries. We have previously reported that patients treated with ACLR had a higher appraisal and adherence to the rehabilitation [29] that contradicts the higher incidence of reinjuries. More focus needs to be put on the rehabilitation protocols, as well as on the return‐to‐sports criteria to evaluate if these can decrease the number of reinjuries. In future analysis of the NACOX study, we plan to analyse the return‐to‐sports criteria that have been reported both from the physiotherapist and the patient. More studies are needed evaluating the risk for knee reinjuries, other than new ACL injuries, after an index ACL injury.

There are several previous studies showing a higher risk for graft rupture and ACL revision surgery after ACLR in young age, return to high level of activity, time to RTS, and graft type in ACLR [2, 13, 15, 23, 32]. In our study, 7% of the patients in the ACLR group had a graft rupture within 2 years, in a time span between 0.9 and 21.4 months after ACLR (Figure 1). We could not identify any risk factor for graft ruptures and ACL revision surgery in the ACLR group. Previous studies show a 10%‒27% [7, 8, 18, 32] risk for graft rupture within 2‒67 months from ACLR. The time span for reinjuries in our study ranges from around 1 month to approximately 24 months after index ACL injury (including graft ruptures), where there is higher incidence after 8 months. In our study, we can see there is a higher number of patients returning to sport level I, compared to level II and level III regarding reinjuries. This is in line with previous reports on higher risk for graft ruptures when returning to high‐level sports. Nevertheless, it is interesting that returning to level III sport had no reinjury.

The seven traumatic reinjuries that occurred before ACLR in the ACLR group may have been avoided if the patients had undergone an early ACLR. Six of these injuries may have been the direct reason for having a late ACLR. Reinjuries, especially meniscus injuries, are known risk factors for the development of knee osteoarthritis [3]. On the other hand, ACLR did not prevent reinjuries as there were more reinjuries after ACLR in our study, including graft ruptures.

One in five patients in our study underwent a knee surgery (excluding primary ACLR) within 2 years from index ACL injury. The incidence of surgeries did not differ between groups, which is in line with the result of the van der Graaff et al. [31] RCT study, but in contrast to other reports showing more surgeries in patients treated without surgery [3, 18, 22]. The reason for undergoing a meniscal surgery can be due to a concomitant meniscus injury at index trauma or due to a new trauma. In our study, we differentiated these numbers to give a clearer picture of the surgical panorama after index ACL injury. We found that 13% in the non‐ACLR group had meniscus surgery, where the majority of the patients (64%) already had the meniscus injury at the baseline MRI, indicating that it was a concomitant injury during the index ACL injury. In the ACLR group, fewer patients underwent a meniscus surgery after ACLR. Still, more than one‐third of the patients in the ACLR group had already undergone meniscus surgery as an additional surgical procedure during ACLR, which is in line with the results reported by van der Graaf [31], where 32% of patients had a meniscal procedure during the ACLR. The most common meniscus procedure in our patients were meniscectomies and meniscal repair. Thus, excluding meniscal procedures done at the same time as the ACLR will underestimate the number of meniscus surgeries in the ACLR population.

There are several strengths with this observational study. We followed patients who were treated according to clinical praxis and by that we can describe the natural corollaries and explore and identify factors that may influence risk of reinjuries or surgeries. This design gives an insight into clinical reality where individualized treatment decisions are made after a shared decision‐making between caregivers and patients [9], thus introducing a selection bias that can be seen as a limitation of the study compared to an RCT design. In addition, patients were included from a geographically spread area, from both private and public clinics increasing the external validity. Still, clinical praxis and healthcare systems differ between countries limiting the generalizability of the study. The inclusion criteria for the NACOX study are quite wide (age 15–40, not excluding contralateral injuries), but representative for the ACL population. This study has extensive follow‐up data in both nonsurgical and surgically treated patients, which many previous studies are missing. In the present analyses, we present results up to 2‐years follow‐up. New injuries and surgeries may occur after that and will be presented in future studies. Another strength is that we separated concomitant injuries at the time of the initial ACL injury from injuries that happened after the initial injury. However, it is hard to categorize reinjuries and symptoms due to the instability of the knee after the initial ACL injury, which could be a limitation. In the present analyses, we included only reinjuries resulting after a specific event that happened during an activity‐related exposure, that is, traumatic injuries, that resulted in increase of knee symptoms or new knee symptoms. Another strength of this study is that we have complete patient‐reported data and verified data from medical charts from all except 11 patients (4%), who did not respond to the questionnaires. Though these 11 patients had no reported new injuries or surgeries when controlling medical charts and data from the National ACL registry. When interpreting the result of our risk analyses, it is important to consider the low number of reinjuries and surgeries, weakening the strength of the statistical analyses that should be carefully interpreted.

The association between reinjuries and surgeries after an ACL injury is complex, multifactorial, and should be seen in context with patient characteristics. Patients in the non‐ACLR group had lower incidence of knee reinjuries and similar amount of knee surgeries (excluding the primary ACLR) compared to patients who had ACLR. As per definition, patients in the ACLR group had at least one knee surgery (the ACLR) that in one‐third of the cases was combined with meniscectomy or meniscal repair. The ACLR group had similar numbers of additional knee surgeries compared to the non‐ACLR group. These results increase the knowledge about side effects and risks for each treatment option that can be used to enhance the shared decision‐making [25]. Further analyses and studies are needed to help clinicians in the decision‐making process for the best treatment of an ACL injury. Parallel analyses of the NACOX cohort are evaluating the outcomes of each treatment strategy including perceived knee function, return to physical activity and sports, and the development of knee osteoarthritis.

## CONCLUSION

In this prospective cohort study, we found that 12% of all patients sustained a knee reinjury and 21% underwent a knee surgery (excluding primary ACLR) within 2 years after the index ACL injury. There was a higher incidence of knee reinjuries in the ACLR group, but no difference in incidence of knee surgeries. Thus, our hypothesis that ACLR will reduce the risk of knee reinjuries or surgeries was not proven, which is interesting and important to consider in the clinical decision of treatment.

## AUTHOR CONTRIBUTIONS

Joanna Kvist and Håkan Gauffin contributed to the study conception and design. Anna S. Selin and Joanna Kvist performed all data collection. Anna S. Selin, Henrik Hedevik, and Joanna Kvist performed the analyses. The first draft of the manuscript was written by Anna S. Selin, and all authors commented on previous versions of the manuscript. All the authors read, edited, and gave their approval to the final manuscript.

## CONFLICT OF INTEREST STATEMENT

The authors declare no conflict of interest.

## ETHICS STATEMENT

The Swedish Ethical Review Authority has approved the NACOX study (Dnr. 2016/44‐31 and 2017/221‐32). This study was performed in line with the principles of the Declaration of Helsinki. Participants received both written and oral information about the study. Informed consent was obtained from all individual participants included in the study.

## Data Availability

Data can be provided on reasonable request.
